# UNHOUSED OLDER ADULTS’ PREFERENCES FOR PROGNOSTIC COMMUNICATION AND ILLNESS-RELATED CARE

**DOI:** 10.1093/geroni/igae098.2640

**Published:** 2024-12-31

**Authors:** Abigail Latimer, Michael Light, Olivia Sasdi, Mohammad Hossain, Debra Moser, Natalie Pope

**Affiliations:** University of Kentucky, Lexington, Kentucky, United States; Harborview Medical Center UW Medicine, Seattle, Washington, United States; University of Kentucky, Lexington, Kentucky, United States; University of Kentucky, Lexington, Kentucky, United States; University of Kentucky, Lexington, Kentucky, United States; University of Kentucky, Lexington, Kentucky, United States

## Abstract

The number of older adults experiencing homelessness (OAEH) is growing. This vulnerable population is exposed to harsh conditions and fragmented healthcare, making even the most treatable health conditions life-threatening. Understanding seriously ill OAEH preferences for prognostic communication and illness-related care are needed research areas. This qualitative study was part of a larger project adapting Ariadne Labs’s Serious Illness Conversation Guide (SICG) for use with OAEH. We interviewed 11 unhoused older adults (≥ 50 years) with a serious illness - mostly men (n = 9) between age 53 and 72 (M = 61). Interview questions were developed corresponding to the model of prognostic communication (i.e., the SICG) with a particular interest in understanding older adults’ 1) communication preferences regarding their illness and prognosis and, 2) goals and values related to their illness and prognosis. We utilized reflexive thematic analysis which included coding and creating transcript summaries and data matrices to help the research team familiarize, condense, and observe patterns within the data. Findings reflect how 1) clinicians should deliver prognosis, 2) OAEH receive prognosis, and 3) OAEH treatment goals and values. Trust and authenticity emerged as essential components of prognostic delivery, both in what and how information is shared. Participants described past communication with healthcare professionals and the impact of these conversations. Illness-related priorities for OAEH included making practical plans for dying and death, leaving a legacy for their children, and maintaining independence. Study findings can inform clinical practice and intervention research to improve how this population receives serious illness communication.

